# Non-feline transmission of sporotrichosis: a systematic review of published cases

**DOI:** 10.1590/S1678-9946202668016

**Published:** 2026-02-16

**Authors:** Pamela Rodríguez-Salgado, Andrés Tirado-Sánchez, Valeria Lyzzete Diaz-Molina, Max Carlos Ramírez-Soto, Flavio Queiroz-Telles, Alexandro Bonifaz

**Affiliations:** 1 Hospital General de México "Dr. Eduardo Liceaga", Servicio de Dermatología, Ciudad de México, Mexico; 2 Universidad Tecnológica del Perú, Facultad de Ciencias, Lima, Peru; 3 Universidade Federal do Paraná, Hospital das Clínicas, Departamento de Saúde Pública, Curitiba, Paraná, Brazil

**Keywords:** Sporotrichosis, Zoonotic diseases, Zoonotic transmission, Non-feline, Sporothrix sp. Sporothrix schenckii

## Abstract

Sporotrichosis is a subcutaneous mycosis caused by species belonging to the genus *Sporothrix*. While zoonotic transmission has been primarily associated with cats, reports have pointed to sporotrichosis transmission by various non-feline animals, albeit infrequently compared with the aforementioned. These reports suggest the potential for zoonotic or environmentally mediated transmission routes with clinical and epidemiological relevance that have yet to be extensively explored. This systematic review organizes the current knowledge on sporotrichosis acquired by non-feline zoonotic transmission. A comprehensive literature review was conducted from January 1980 to March 2025, leveraging prominent databases like PubMed, SciELO, Web of Science, and EBSCO to identify cases of sporotrichosis transmitted by non-feline animals. A total of 78 cases of sporotrichosis transmitted by non-feline animals were identified in 26 articles. Most cases (76%) were transmitted by vertebrate animals, primarily dogs, whereas 24% were transmitted by invertebrates, such as mosquitoes. Lymphocutaneous presentation predominated among clinical manifestation in 80.7% of cases. Most frequently isolated species was *Sporothrix schenckii,* whereas *Sporothrix brasiliensis* was isolated only in infections caused by vertebrate animals. Most patients were young adult males, mainly related with hunting activities. Itraconazole was the most frequently used treatment. Sporotrichosis transmission route via non-feline animals is significant and frequently underestimated. Further research is needed to improve our understanding of the transmission mechanisms, focusing on distinguishing between direct zoonotic transmission and environmental exposure mediated by animal contact. Such enhanced understanding is crucial for improving diagnostic procedures in hyperendemic regions and strengthening epidemiological surveillance.

## INTRODUCTION

Sporotrichosis is a subcutaneous mycosis caused by dimorphic species belonging to the genus *Sporothrix*, which comprises at least seven species, including *Sporothrix schenckii* (sensu stricto), *Sporothrix brasiliensis*, *Sporothrix globosa*, and *Sporothrix mexicana*, which are responsible for most cases transmitted to humans^1–3^. These microorganisms typically reside in soil, decaying vegetation, and organic matter and are capable of infecting humans and animals via traumatic inoculation with thorns, splinters, hay, sphagnum moss, and conifer needles^4–6^.

Cutaneous disease is clinically classified into three different forms: lymphangitic sporotrichosis, fixed cutaneous, and disseminated (cutaneous and visceral)^4–6^. Lymphangitic sporotrichosis is the most common clinical form, characterized by the appearance of nodular lesions that run upward along the lymphatic system. The fixed form presents lesions localized to the inoculation site, whereas the disseminated form is associated with immunosuppressive factors and can affect various organs including the skin, lungs, bones, and central nervous system. The pulmonary form is caused by aspiration of the fungal spores^7–9^.

For many years, sporotrichosis has been considered a disease associated with agricultural or forestry activities, but in the 90's this paradigm changed due to reports of zoonotic outbreaks, particularly in Brazil, where cats were identified as the main vectors of the disease, associating fungus transmission with scratches or bites from these animals^1,10,11^.

This transmission route has caused such an impact that it has reached hyperendemic proportions with thousands of published reports, to the point of being considered an emerging zoonosis, in addition to representing an epidemiological and public health challenge^12–14^. For this reason, medical and epidemiological attention has focused on feline transmission, displacing other less frequent forms of zoonotic transmission associated with non-feline animals, regardless of whether these transmission routes are truly zoonotic or environmentally mediated, including those related to birds^15^, dogs^6,16^, armadillos^2,17^, mosquito^18^, bats^13^, rodents^3^, ants^19,20^, spiders^12^, and squirrels^21^.

Forms of sporotrichosis transmitted by non-feline animals tend to be reported as isolated cases and are frequently underestimated. The current literature lacks a systematic analysis that consolidates the mechanisms of non-feline zoonotic transmission, so the true relevance of this transmission route, its geographical distribution, the vectors frequently involved and the clinical manifestations that guide its diagnosis and treatment in the absence of the classic history of contact with cats are unknown^18–20^. Likewise, this study can help us recognize associated risk factors and establish effective preventive and therapeutic strategies, and also increase the reports of sporotrichosis transmitted by animals, especially in hyperendemic areas^16,21,22^.

Recognizing the different *Sporothrix* vectors is essential for designing epidemiological surveillance programs, mainly targeting populations at high risk of sporotrichosis like farmers, hunters, forestry workers, or people with regular contact with wild animals and insects^23–25^.

Thus, this systematic review integrated the existing knowledge on published cases of non-feline-transmitted sporotrichosis, including direct animal or indirect environmental transmission, and focused on the clinical, microbiological, and therapeutic dimensions of the condition.

## MATERIALS AND METHODS

We conducted a bibliographic search on multiple databases (including PubMed, SciELO, Web of Science, and EBSCO) from January 1980 (the first reported onset of zoonotic outbreaks was in 1990) to March 2025 to identify all case reports and case series describing patients with sporotrichosis transmitted by non-feline vectors. The resulting systematic review was conducted according to the Preferred Reporting Items for Systematic Reviews and Meta-Analyses (PRISMA) guidelines. Inclusion criteria included patients with sporotrichosis confirmed by positive *Sporothrix* culture from tissue or clinical specimens transmitted by non-feline vectors. Patients were classified according to sporotrichosis variety (cutaneous/systemic), vector (animal/insect), and *Sporothrix* species isolated from tissue or clinical samples (skin biopsy/swab). Review articles and case series lacking information on infection site or the vector involved were excluded. The search was broadened by manually reviewing the references of selected articles to identify additional studies. MESH terms and Boolean operators used included (extracutaneous AND Sporotrichosis) OR (cutaneous AND Sporotrichosis) OR (zoonotic transmission AND Sporotrichosis) OR (Epidemiological AND Sporothrix) OR (Cutaneous and Sporothrix) OR (Disseminated AND Sporothrix) OR (hypersensitivity AND Sporothrix) OR (dog AND Sporothrix) OR (cattle AND Sporothrix) OR (horse AND Sporothrix) OR (goat AND Sporothrix) OR (rat AND Sporothrix) OR (armadillo AND Sporothrix) OR (swine AND Sporothrix) OR (mule AND Sporothrix) OR (donkey AND Sporothrix) OR (camel AND Sporothrix) OR (fox AND Sporothrix) OR (dolphin AND Sporothrix) OR (bird AND Sporothrix) OR (primate AND Sporothrix) Human. The main search strategy phrase included the following keywords: "Zoonotic transmission OR zoonotic transmitted AND sporotrichosis AND diagnosis AND treatment"

### Quality assessment of articles

Quality assessment of the included cases used the Joanna Briggs Institute's critical appraisal tools^26^. Inclusion criteria, sample size, participant descriptions, and context of the selected studies were reviewed. Two researchers (PRS and ATS) conducted the search independently, thereby reducing bias. A third researcher (AB) evaluated the quality of the selected studies. Quality assessments were performed using different tools depending on the study design. Each tool was modified to obtain a numerical score. Quality assessment of our systematic review used the AMSTAR 2 criteria, showing a low level of compliance. The inclusion of only case reports and observational studies, without quantitative data, made a meta-analysis unfeasible.

### Inclusion criteria

Only articles in English, Spanish, and Portuguese with complete details on the clinical presentation, diagnostic, and treatment methods for each patient were included in this systematic review. Cases were grouped and analyzed within each category (vertebrate-borne or invertebrate-borne disease) based on sex, age, level of endemicity (hyperendemic and endemic), time and location, primary site of infection and sites of spread, presence of comorbidities, outcomes, microbial data, diagnostic and treatment methods, and prognosis.

### Exclusion criteria

Title and abstract screening excluded studies if they did not involve human subjects, did not mention contact with animals or vectors, were unrelated to sporotrichosis, or lacked clinical case information. Narrative reviews, editorials, experimental studies without clinical correlation, and conference abstracts without full case data were excluded. These criteria were applied to ensure the inclusion of only relevant clinical reports.

### Statistical analysis

To evaluate the independent association between sporotrichosis variety and the clinical, microbiological, and therapeutic response characteristics of the patients analyzed, we compared cases with vertebrate-borne and invertebrate-borne sporotrichosis using univariate analysis with chi-square and Student's t-tests. Statistical analysis was performed on SPSS software (version 24, SPSS, Chicago, IL, USA).

## RESULTS

**Figure 1:**
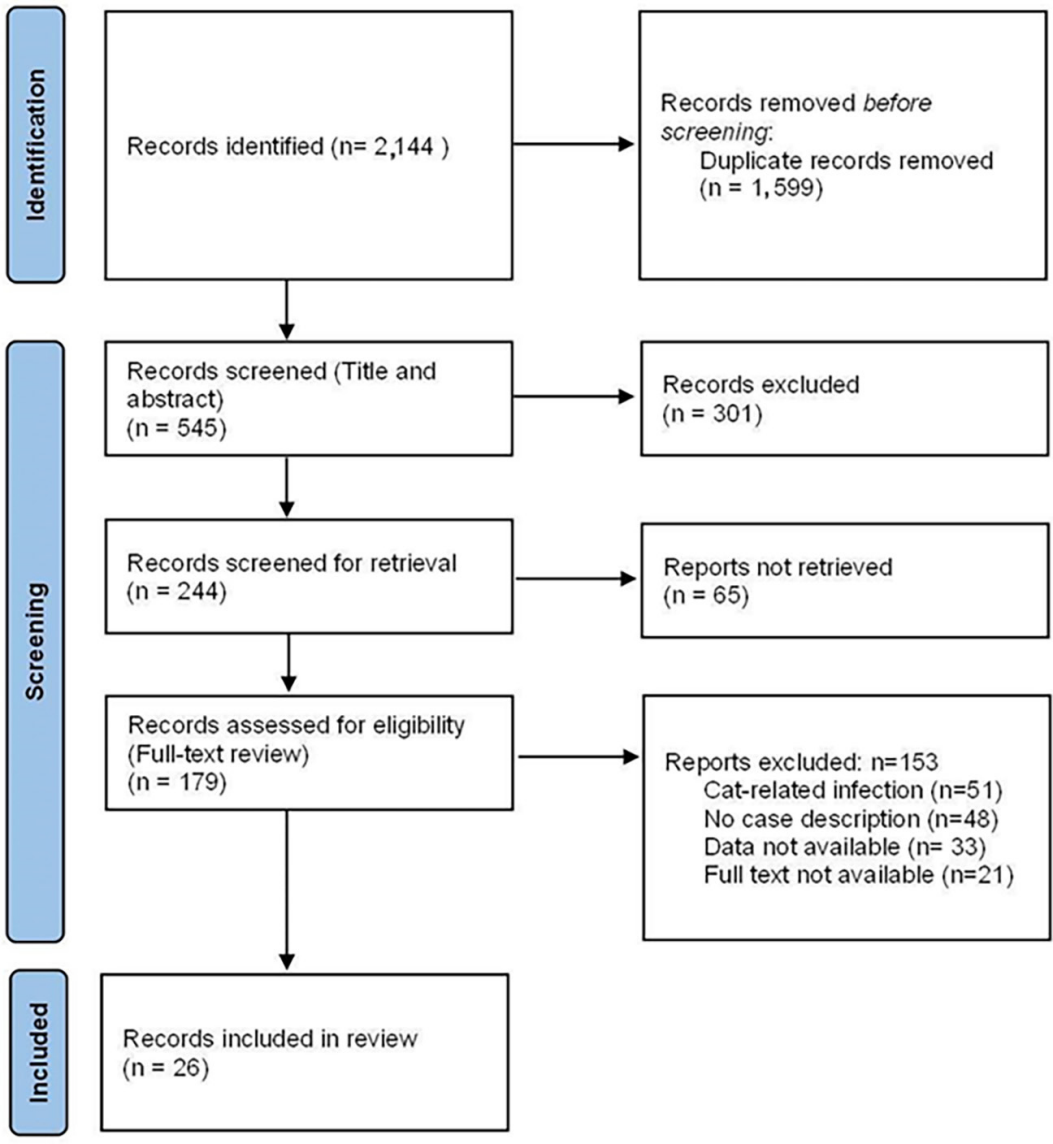
Flowchart of the study selection and inclusion process.

We identified 2,144 publications using this systematic search. After removing duplicates, we assessed 545 articles for eligibility based on title and abstract. Full-text analysis was performed in 244 papers, of which 26 met the established inclusion criteria (Figure 1).

**Figure 2:**
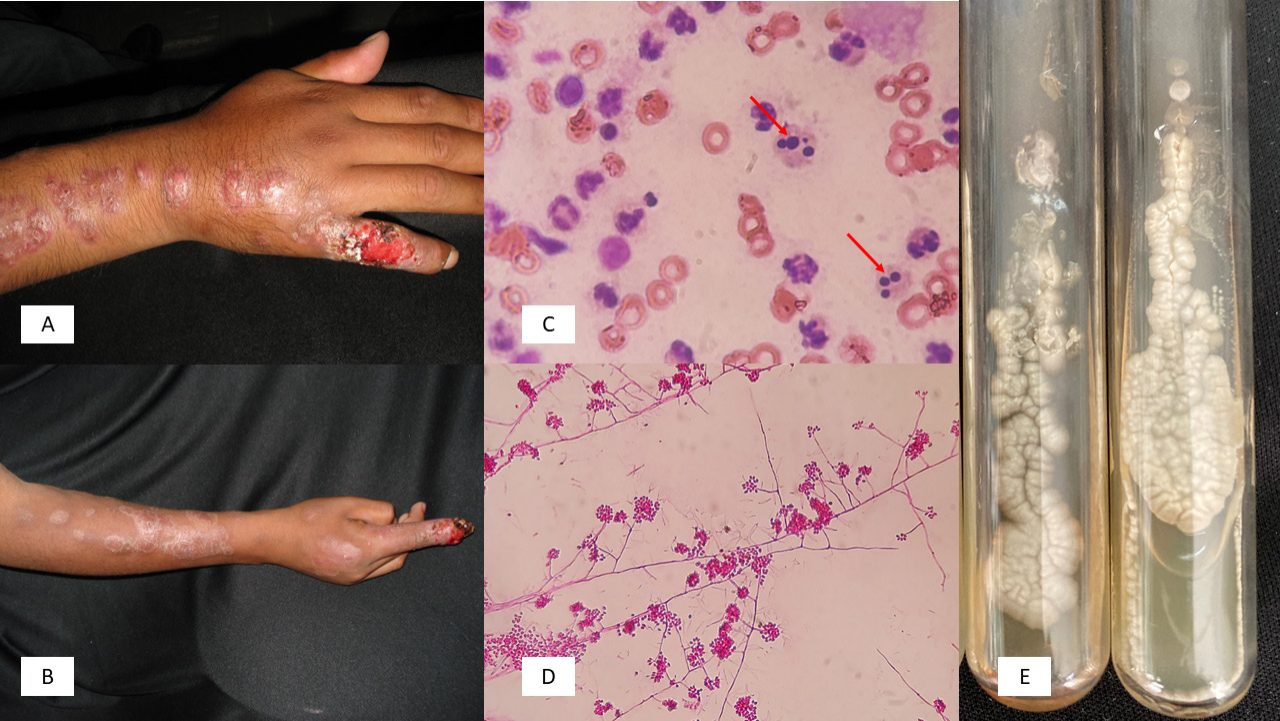
(A) Sporotrichosis after a squirrel scratch; (B) Sporotrichosis after an armadillo capture; (C) Exudate smear, arrows indicate *S. schenckii* yeasts (Giemsa, 100x); (D) Filamentous phase with sympudolic microconidia of *S. schenckii* (erythrocin, 40x); (E) *S. schenckii* culture, filamentous phase.

A total of 78 cases from 26 scientific articles were included (Table 1). Brazil (48 cases) and Mexico (10 cases) reported most cases. The youngest patient was 1 year and 9 months old (insect bite transmission), and the oldest was 68 years old (vampire bat bite transmission). Mean patient age was 28.65 ± 20.76 years. Most cases where age could be determined (n=20) were 18 years or younger (n=9, 45%), followed by those between 19 and 30 years (n=3, 15%), between 31 and 50 years (n=3, 15%), or over 51 years (n=5, 25%). Age was not reported in 58 cases. Of the 35 cases where sex was reported, 80% of the population included in the studies were male (28 cases), whereas 20% were female (7 cases). The patient's sex was not reported in 43 cases. *S. schenckii* was found in 46% of cases (n=36), followed by *S. brasiliensis* (n=26, 33%) and *S. globosa* (n=4, 5%). In 12 cases (16%), it was not possible to determine the species involved. Vertebrate and invertebrate vectors were associated with human sporotrichosis (Figure 2). Dogs were the most frequently associated species with the former (31 cases, 40%), and mosquitos with the latter (6 cases, 8%), and arthropods were generally associated in up to 24% of the cases studied (n=19) (*P=*.001). In the examined cases, the primary infection route was most frequently a bite or scratch, most commonly inflicted by canines. Table 2 compares the characteristics of the cases according to the vector involved. Lymphocutaneous form (n=63, 80.7%) was the most frequent clinical variety, followed by the fixed form (n=8, 10%) and disseminated cutaneous (n=4, 5%); the clinical variety was not reported in 3 cases. The most affected topography was the left upper extremity (n=13, 16%), followed by the right upper extremity (n=7, 9%); however, the affected region remained undetermined in 50 cases (64%). The occupations/professions of the 78 patients were distributed as follows: student (n=12, 16%); hunter (n=11, 14%), homemaker (n=4, 5%); service worker and salesperson (n=4, 5%); fisherman (n=3, 4%); gardener (n=1, 1%); farmer (n=1, 1%); not informed (n=42, 54%).

**Table 1:** Clinical characteristics and outcome of patients with non-feline zoonotic sporotrichosis

Article	Country	(*n*)	Sex	Age (years)	Presentation	Infection site	Infection source	Transmission route	Treatment	Isolated	Response
Rodrígues *et al.* ^2^	BRA	1	M	51	LC/pulmonary	Upper extremity	Armadillo	Scratch	ITZ	*S. schenckii*	Clinical cure
Hayfron *et al.* ^3^	USA	1	F	7	C/CL	Upper extremity	Rat	Bite	ITZ	*S. schenckii*	Clinical cure
Bonifaz *et al.* ^5^	MEX	4	M	8-12	LC	NR	Squirrel: 3; rat: 1	Scratch or bite	KI + ITZ	*S. schenckii*	Clinical cure
Oliveira Ramos *et al.* ^6^	BRA	1	F	NR	F/C	Upper extremity	Dog	Scratch or bite	AMB + ITZ	*S. schenckii*	Clinical cure
Bittencourt *et al.* ^8^	BRA	3	NR	NR	NR	NR	Dog	No trauma reported	ITZ	*S. globosa*	Clinical cure
Tang *et al.* ^9^	MYS	3	F	43	LC	Upper extremity	Fish	Fish handling	ITZ	*S schenckii*	Clinical cure
Tang *et al.* ^9^	MYS	10	M	24	F	Upper extremity	Insect	Bite	TRB	*S schenckii*	Clinical cure
Tang *et al.* ^9^	MYS	1	M	23	F	Upper extremity	Fish	Fish handling	EB	*S schenckii*	Clinical cure
Haddad *et al.* ^10^	BRA	1	M	18	LC	Upper extremity	Fish	Fishbone sting	KI	*Sporotrhrix sp*	Clinical cure
Rojas-Padilla *et al.* ^12^	MEX	1	M	13	LC	Upper extremity	Spider	Bite	KI	*S schenckii*	Clinical cure
Martínez Rodríguez *et al.* ^13^	MEX	1	F	68	LC	Upper extremity	Bat	Bite	KTZ + TMP-SMX	*S schenckii*	Clinical cure
Carrada-Bravo *et al.* ^14^	MEX	1	M	1.9	FS	Face	Insect	Bite	KI	*Sporotrhrix sp*	Clinical cure
Fichman *et al.* ^15^	BRA	1	M	35	F/C	Trunk	Cockatiel	Scratch	ITZ	*S. schenckii*	Clinical cure
Gallo-Echeverri *et al.* ^16^	COL	1	M	64	F	Upper extremity	Dog	Scratch	ITZ	*S. globosa*	Clinical cure
Alves *et al.* ^17^	BRA	1	M	18-49	LC/F	Upper extremities	Armadillo	Scratch	ITZ	*S. schenckii*	Clinical cure
Moore *et al.* ^18^	USA	1	F	9	LC	Upper extremity	Mosquito	Bite	ITZ	*Sporothrix sp*	Clinical cure
Miller *et al.* ^20^	USA	1	M	54	LC	Upper extremity	Ant	Sting	ITZ	*Sporotrhrix sp*	Clinical cure
Saravanan *et al.* ^21^	USA	1	M	23	LC	Upper extremity	Squirrel	Bite	ITZ	*S. schenckii*	Clinical cure
Barba-Borrego *et al.* ^22^	MEX	1	M	12	Bilateral LC	Upper extremity	Gopher	Bite	KI	*S. schenckii*	Clinical cure
Nahal *et al.* ^23^	BRA	5	NR	NR	LC	Left arm: 1/ NR:4	Insect: 4; fish: 1	Bite or sting	NR	*S. schenckii*	Clinical cure
Jirawattanadon *et al.* ^25^	THA	3	NR	NR	LC	Lower extremities	Infect: 2; NR: 1	Bite	ITZ	*Sporothrix sp.*	Clinical cure
Griffin *et al.* ^28^	PER	1	M	34	LC	Upper extremity	Insect	Bite	ITZ	*S. schenckii*	Complete cure
Charles *et al.* ^31^	USA	1	F	57	C (PG-like)	Upper extremity	Arthropod	Bite	ITZ	*S. schenckii*	Improve
Mesquita *et al.* ^32^	BRA	26	NR	NR	LC	NR	Dog	Bite or scratch	ITZ	*Sporotrhrix sp*	Clinical cure
Mata-Essayag *et al.* ^33^	VEN	6	NR	NR	LC	NR	Insect:5; Opposum:1	Bite	KI	*Sporotrhrix sp*	Effective
Perez-Elizondo *et al.* ^34^	MEX	1	M	13	LC	Upper extremity	Squirrel	Bite	ITZ	*S. schenckii*	Clinical cure
Perez-Elizondo^35^	MEX	1	F	8	F	Upper extremity	Squirrel	Bite	TRB	*S. schenckii*	Clinical cure
Beer-Romero *et al.* ^36^	VEN	1	M	16	LC	Upper extremity	Fish	Knife wound	NR	*NR*	NR

NR = not reported; BRA = Brazil; USA = United States of America; MEX = Mexico; MYS = Malaysia; COL = Colombia; THA = Thailand; PER = Peru; VEN = Venezuela; LC = lymphocutaneous sporotrichosis; FS = facial sporotrichosis; F = fixed sporotrichosis; DS = disseminated sporotrichosis; C = cutaneous sporotrichosis; ITZ = itraconazole; EB = excisional biopsy; KTZ = ketoconazole; TRB = terbinafine; TMP-SMX = trimethoprim-sulfamethoxazole; K = potassium iodide; AMB = amphotericin B; PG-like = pyoderma gangrenosum like.

**Table 2:** Demographics and clinical characteristics from patients with Zoonotic non-feline transmitted sporotrichosis

Characteristic		Vertebrate-transmitted	Invertebrate-transmitted	aPR (95%CI)*****	*P* Value
No. (%) of patients		59 (76%)	19 (24%)		
Age, y		28.65 ± 20.76			
	Mean (SD)	29.3 ± 21	27.4 ± 21.91	0.88 (0.74-0.97)	.035*
	Range	7-68	1-57		
Sex, (%) of the patients M/F		28 (80)/7 (20)			
	Male	24 (86)	5 (71)	1.22 (1.05-1.43)	.018**
	Female	4 (14)	2 (29)	Ref.	
Sporotrichosis clinical form, N° (%) of patients***					
	Lymphocutaneous	51 (86)	15 (79)		.01
	Fixed cutaneous	4 (7)	3 (16)		
	Disseminated	1 (2)	1 (5)		
Level of endemicity					
	Hyperendemic****	53 (90)	7 (37)	1.82 (1.43-2.5)	.012
	endemic	6 (10)	12 (63)	Ref.	
Organism, N° (%)					
	*Sporothrix schenckii*	26 (44)	8 (42)		NS
	*Sporothrix brasiliensis*	26 (44)	0 (0)		
	*Sporothrix globosa*	4 (7)	0 (0)		
	*Sporothrix sp*	2 (3)	11 (58)		
	Not reported	1 (2)	0 (0)		

aPR = adjusted prevalence ratio; SD = standard deviation;

* t-Student;

** Fisher's exact test;

*** 3 cases not reported in clinical form;

**** Hyperendemic: Brazil, an area known to have a high rate of sporotrichosis. Other hyperendemic areas include Mexico, Peru, China, and Colombia;

***** Poisson regression test.

Only one case (1.3%) reported data on comorbidities. This 57-year-old patient presented obesity and asthma, and developed ulcer following an arthropod bite. She may have experienced an element of iatrogenic immunosuppression due to steroid use, given the initial presumptive diagnosis of pyoderma gangrenosum. Comorbidities could not be determined in 46 patients (59%). The remaining 31 cases (39.7%) had no mention of comorbidities at all. This lack of systematic reporting limits our ability to assess potential associations between underlying health conditions and clinical presentation.

Itraconazole was the most commonly used treatment (n=53, 68%), with doses ranging from 100-200mg daily every 12 h, depending on infection severity, and a mean duration of 114 ± 47 days (range 60-270 days). Only one patient underwent surgical excision. Other less frequently used but successful treatments included potassium iodide, terbinafine, ketoconazole, and amphotericin B.

## DISCUSSION

Our systematic review identified 78 cases of zoonotic sporotrichosis transmitted by non-feline animals in a total of 26 articles. Most cases (76%) were transmitted by vertebrate animals, primarily canines (40%), whereas invertebrates, primarily mosquitoes, were responsible for 24%. These percentages are significant due to the lack of information on differences between both groups (vertebrate and invertebrate vectors) regarding clinical, microbiological, and epidemiological characteristics.

**Figure 3:**
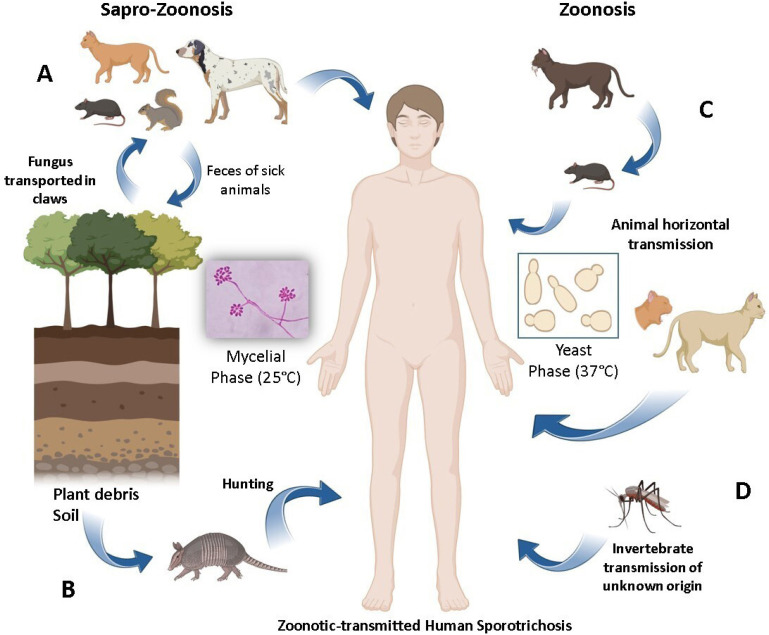
Transmission mechanisms of zoonotic sporotrichosis. Zoonotic transmission may be related to sapro-zoonotic mechanisms involving the fungus in its mycelial phase, and exclusively zoonotic transmission mechanisms involving the fungus in its yeast phase: (A) The agent is usually found in the soil and decomposing plant matter in its mycelial phase, which can be transported (without causing disease) by different animals that can inoculate the fungus through deep scratches or eliminate it through feces on the ground; (B) Activities like hunting can increase the risk of transmission by scratches or bites; (C) Contact with the exudate from skin lesions of sick cats or dogs can transmit the infection horizontally to other animals, including those of the same species and small species (mice, rats, squirrels). Likewise, the infection can reach humans by this route via scratches, bites, and secretions from sick animals. Although rare, human-to-human transmission can occur; (D) Infection may be related to invertebrates that can inoculate the agent through bites or punctures, although it is unclear whether the transmission is sapro-zoonotic or zoonotic, as these animals come into contact with sick animals.

According to our review, most cases involved sapronotic transmission due to contaminated, but not infected, animal claws that, by inoculating the fungus in its mycelial phase through scratches, induce the disease in humans. In addition to the sapronotic (or sapro-zoonotic) route, sporotrichosis has also been observed after bites and scratches from various animals, which can also develop sporotrichosis. In cases of sporotrichosis involving animal contact, the infection in humans may result from direct zoonotic transmission or indirect environmental exposure mediated by animals^4–6^ (Figure 2). The association between invertebrate animals (primarily insects) and the development of sporotrichosis in humans has been documented^27^. This transmission occurs because insects act as indirect vectors, but it is unclear whether they inoculate the infectious agent or merely facilitate its entry through the bite or sting^14^ (Figure 3).

Epidemiologically, patients with sporotrichosis transmitted by invertebrates were younger than those with sporotrichosis transmitted by vertebrates, which was a statistically significant finding that could lead to interpretation bias due to the sample size. The youngest patient in the entire series was a 1 year 9 month old child who developed fixed cutaneous sporotrichosis after an insect bite in Mexico and was treated with potassium iodide at a dose of 1 gram per day, achieving complete recovery from the condition after 2 months. This could reflect a vulnerability in the pediatric population due to an underdeveloped immune system, but it also shows the effectiveness of therapeutic options in pediatric cases^14^.

Regarding sex, 80% of patients were male, mainly infected by vertebrate animals (86%) compared with invertebrates (71%). This difference could be explained by the different activities related to occupational and recreational exposure: hunting, fishing, interaction with animals in rural areas^28^. As for cat-transmitted sporotrichosis, particularly in Brazil, the most affected group are adult females, frequently housewives, due to contact with domestic animals and activities such as caring for sick cats^11^. Interestingly, several studies have shown that healthy cats from hyperendemic regions rarely harbor *Sporothrix* spp. in their oral, nasal, or paw mucosae, suggesting that even in these settings, environmental contamination may play a larger role than direct animal-to-human transmission. This finding challenges the idea that asymptomatic felines act as persistent infection reservoirs and supports the notion that infected animals may primarily contaminate the environment rather than serve as constant sources of zoonotic transmission. This underscores the complexity of attributing causality in suspected zoonotic cases and the need for molecular studies to confirm true reservoirs^8,23^.

Although sociodemographic and occupational data were collected, their direct correlation to specific vectors remains challenging due to limited information in the original case reports^2^. But certain patterns, such as the predominance of young male hunters or fishermen in vertebrate-associated cases and children in insect-related cases, suggest potential associations between exposure type and transmission route^2,5^.

Clinically, the lymphocutaneous presentation was more common in both groups. Fixed and disseminated cutaneous forms were also reported, including one case of sporotrichosis mimicking an atypical pyoderma gangrenosum in a 57-year-old woman from the United States. She presented with a lesion following an arthropod bite that developed into a painful, purplish ulcer with undermined edges, initially diagnosed as neutrophilic dermatosis. As clinical improvement was lacking, *S. schenckii* was isolated and responded favorably to itraconazole. Significant medical history included asthma and obesity, and she was initially treated with systemic corticosteroids, underscoring the mimetic capacity of this mycosis, particularly in immunocompromised patients^29^. This highlights the importance of recognizing the clinical variability of the disease, and of suspecting it in the differential diagnosis of chronic skin lesions with a history of contact with animals or insect bites.

Regarding geographic distribution, 90% of cases were reported in hyperendemic regions (Brazil and Mexico), coinciding with the cases reported due to the zoonotic emergence of cat-borne sporotrichosis, highlighting the presence of alternate vectors in these areas. This epidemic generated greater diagnostic awareness, epidemiological surveillance, and improvements in identifying sporotrichosis in humans, increasing not only the identification of cat-associated cases but also those transmitted by different vectors in the same environment. In other words, it favored the identification of other transmission routes. *S. schenckii* was the most common species in both groups. *S. brasiliensis* was found exclusively in infections associated with vertebrate animals, whereas cases involving invertebrates were most often reported as *Sporothrix* sp. without species-level identification. This may reflect true biological differences or (more likely) limitations in diagnostic resources, particularly molecular tools, in certain settings. Thus, no definitive correlation between vector type and fungal species can be established based on current data.

However, hyperendemic regions such as Brazil have greater access to molecular tools that allow species differentiation^30^. In this context, *S. brasiliensis* is the predominant species in cat-associated infections, accounting for more than 90% of human cases. Thus, species distribution may depend on both the vector and the geographic context and diagnostic capacity of the area in which the cases are identified.

As for treatment, itraconazole was the most commonly used option, showing an adequate therapeutic response in most cases. Other treatments such as potassium iodide, terbinafine, ketoconazole, and amphotericin B were used in other cases, all showing adequate improvement. The reported therapeutic diversity reflects the need to individualize treatment according to resource availability and the good response when starting treatment after identifying the condition based on suspected zoonotic transmission and transmission by non-feline animals. Itraconazole efficacy has been widely documented in cases of feline-transmitted sporotrichosis and it constitutes the treatment of choice, showing cure rates over 90%^30^.

Although ketoconazole was employed in some of the earlier cases included in this review, its use is currently discouraged in international guidelines due to hepatotoxicity and lower efficacy compared to newer antifungal agents. In countries like Mexico, however, ketoconazole remains commercially available in pharmacies and has not been officially withdrawn. Its low cost and accessibility contribute to its continued use in some public and private healthcare settings, particularly in resource-limited contexts where itraconazole or other alternatives may not be readily accessible^5,6^.

Some Latin American regions have reported transmission by various animals such as squirrels, dogs, insect bites, and a single case transmitted by fish^30–36^. The latter is notable because it is still unclear whether the fungus can survive in aquatic conditions or if it is a subsequent infection due to handling in endemic areas. For example, a report from Guatemala (Lago de Ayarza), considered a hyperendemic area for sporotrichosis,^31^ states that 24 out of 53 cases (45%) developed the disease after handling fish. We did not include this report in the review due to the unclear direct relation, but it is important to keep this possible transmission route in mind^31^.

Importantly, not all reported cases involving vertebrate animals necessarily represent direct zoonotic transmission. In several of the cases involving canines, it is unclear whether the animals themselves were actively infected or whether they were mechanical vectors for *Sporothrix* species acquired from contaminated environments, particularly from close contact with infected cats^6,16^. In this scenario, two plausible transmission mechanisms must be considered: (a) direct zoonotic transmission from a diseased animal with active fungal lesions^16^, and (b) environmental transmission through contaminated claws or fur, without the animal itself manifesting clinical disease.^6^ Such a distinction is relevant because in hyperendemic regions like Brazil, the environment may be heavily contaminated with fungal propagules shed by sick animals, particularly cats, allowing for indirect transmission^37^. The possibility that dogs or other animals act as passive carriers of environmentally distributed *Sporothrix* conidia should therefore be carefully considered when interpreting these cases^6,37^.

In addition to direct and indirect animal-mediated transmission, environmental exposure via contaminated fomites must also be considered^38^. *Sporothrix* spp. are known to persist in organic material and contaminated surfaces, and rare cases of transmission by non-animal vectors like tattoo instruments have been reported in the literature^39^. These examples emphasize that transmission may occur in the absence of direct animal contact and that environmental contamination can be a critical factor, especially in hyperendemic regions.

Similarly, armadillo hunting has long been identified as a risk factor for sporotrichosis in regions of Brazil^2,17^. However, this association is likely related to environmental contamination during handling or exposure to fungal reservoirs in soil or vegetation disturbed during hunting, rather than to zoonotic transmission from the animal itself. Consequently, while animal involvement is frequently reported, it may reflect environmental exposure rather than a true zoonotic source^30,32^.

Importantly, cases involving insect bites should not be classified as zoonotic transmission in the strict sense^20^. Unlike vertebrates, insects do not develop active *Sporothrix* infections and are unlikely to serve as biological reservoirs. Rather, their role is likely mechanical or environmental, transporting fungal elements from contaminated surfaces to human hosts. This pattern is more consistent with environmental transmission mediated by invertebrate vectors^20^. Similar mechanisms have been described with other environmentally distributed fungi such as species of the *Entomophthorales* order, where insects act as carriers without being true pathogen hosts^40^.

Study limitations include the scarcity of studies on sporotrichosis transmitted by non-feline vectors which limits definitive conclusions. The available reports were heterogeneous regarding the clinical, microbiological, and therapeutic data presented, which complicated data synthesis by discarding incomplete articles. Finally, the speculative nature of the findings requires further studies focused on transmission mechanisms to clarify whether transmission by non-feline vectors is supported or is a misinterpretation of a sapronosis or transmission indirectly mediated by feline vectors. Another possible limitation is the low quality of the reports and case series analyzed, which could result in possible bias because evidence certainty is low.

Additionally, a significant proportion of cases lacked essential information such as patient comorbidities and immunosuppressive status, which further restricted our analysis and hindered the identification of relevant clinical or epidemiological associations.

## CONCLUSIONS

Our current knowledge regarding sporotrichosis transmitted by non-feline vectors is limited, primarily due to the lack of comprehensive data in many cases, including details such as age, sex, specific vectors, and anatomical region. To further broaden this knowledge, future studies should focus on the transmission mechanisms, risk factors for acquiring the disease, and entry routes. These are essential for understanding not only the nature of the infection but also for establishing appropriate preventive, diagnostic, and therapeutic measures.

## Data Availability

The complete anonymized dataset supporting the findings of this study is included within the article itself.
